# Husband’s knowledge on partner’s reproductive rights and its associated factors in central Ethiopia: a community-based cross-sectional study

**DOI:** 10.1017/S1463423626100917

**Published:** 2026-02-19

**Authors:** Selamawit Nigatu, Fikremariam Endeshaw, Mangistu Abera, Wudit Wasu, Zigijit Azene, Tigist Derebe, Aberash Beyene Derribow

**Affiliations:** 1 Midwifery, Wachemo University, Ethiopia; 2 Midwifery, Wolkite University, Ethiopia; 3 Midwifery, Bahir Dar University, Ethiopia

**Keywords:** Ethiopia, husband’s knowledge, partner, reproductive health rights, Wolkite

## Abstract

**Background:**

Women’s sexual and reproductive rights are crucial for achieving gender equality and promoting women’s rights. Across East Africa, there are limited studies about husbands’ knowledge of their partner’s reproductive rights and their associated factors. Hence, this study aimed to assess husbands’ knowledge of partners’ reproductive health rights and associated factors in Central Ethiopia.

**Methods:**

A community-based cross-sectional study was conducted from March 15 to April 30, 2023, using multi-stage stratified sampling. A structured, interviewer-administered questionnaire was utilized to gather the data, and then SPSS version 26 was employed for analysis. Statistical significance was declared at a *p*-value < 0.05.

**Results:**

The overall good knowledge of partners’ reproductive health rights was found to be 47.8% (95% CI: 43.8, 51.8). Age of the husbands 25–35 years (AOR: 2.7; 95% CI: 1.10, 6.6), below primary educational status (AOR: 2.4; 95% CI: 1.3,4.3), primary educational status (AOR: 5.98; 95% CI: 3.10, 11.4), secondary educational status (AOR: 2.1; 95% CI: 1.01, 4.3), above secondary education status (AOR: 8.0; 95% CI: 4.3, 15.2), discussion with a partner (AOR: 3.2; 95% CI: 2.0, 5.2), and vehicle as a means of transport (AOR: 3.3; 95% CI: 2.2, 4.9) were statistically significant for good husbands’ knowledge of partners reproductive health rights.

**Conclusion:**

These findings indicate that more than half (52.2%) of the study participants had a poor understanding of their partners’ reproductive rights. Therefore, counselling and education should be offered to husbands to ensure equitable access to health services and to disseminate information on reproductive rights, particularly targeting young men.

## Introduction

Reproductive rights are the freedom to choose about reproduction without discrimination, coercion, or violence and include twelve basic human rights such as life, liberty, security, privacy, equality, consent to marriage, health, access to education, family planning, and scientific progress based on the International Conference on Population and Development (ICPD) frame work (Assembly, 1948; World Health Organisation, 2014; World Health Organisation, 2019). The United Nations has prioritized gender equality and the empowerment of women and girls as Goal 5 to ensure universal access to sexual and reproductive health rights, aiming to achieve the Sustainable Development Goals by 2030 (Germain, 2014). In the mid-1990s, the ICPD held in Cairo marked a shift by recognizing men’s shared responsibility in advancing women’s reproductive rights (Barroso, 2014).

Globally, approximately 30% of women experience reproductive rights violence, particularly by their intimate partner (World Health Organisation, 2013). Despite these global efforts, violence against women’s reproductive rights remains a significant challenge, particularly regarding physical and sexual access to reproductive education, services, and contraceptive use (World Health Organisation, 2019). The abuse of reproductive rights by these partners presents a public health concern, particularly in African countries where reported rates range from 20% to 70% (Beyene, 2015; Quarcoo and Tarkang, 2019). Many women believe that one of the main causes of intimate partner violence (IPV) is a lack of knowledge and assistance about sexual and reproductive health rights and services (Adinew *et al.*, 2013a). Further, intimate partner sexual violence among married women is one of the root problems of reproductive rights (Simona and Likando, 2025). The women’s reproductive rights violations are attributed to husbands’ insufficient knowledge, lack of reproductive education, and lack of spousal discussions on reproductive issues (Mayer *et al.*, 2025). Studies across countries have consistently revealed that husbands generally have low levels of knowledge regarding reproductive health rights and are less engaged in their partners’ reproductive health care (García-Moreno, 2005; Barot, 2015).

In many developing countries, men influence women’s access to resources, and studies show that husbands’ involvement in reproductive rights improves women’s access to maternal health care services (Chernet and Cherie, 2020). The Ethiopian Federal Ministry of Health (FMOH), in line with the WHO, has set seven strategic directions to prevent women’s reproductive and other human rights violations (García-Moreno *et al.*, 2005). A 2013 WHO study found Ethiopia had the highest rate of intimate partner reproductive rights violations among ten countries, with 53.7% experiencing sexual, physical, or both violations within one year and 70.9% over their lifetime (García-Moreno *et al.*, 2013). The study found that 29.6% of intimate partners violated their reproductive health rights in the Southern Nations, Nationalities, and Peoples’ Region (SNNPR) of Ethiopia (Mohammed *et al.*, 2021b).

The evidence revealed that limited knowledge among husbands about their partners’ reproductive health rights is associated with women’s increased risk of physical violence, which in turn correlates with adverse reproductive health outcomes (Asratie *et al.*, 2024; Jemberie *et al.*, 2024b). Studies conducted in Ethiopia indicated that husbands’ knowledge of their partners’ reproductive health rights ranged from 48.3% to 50.6% (Nur *et al.*, 2020; Diamond-Smith *et al.*, 2024). Insufficient knowledge among husbands regarding their partners’ reproductive health rights contributes to delayed or absent care-seeking, unmet contraceptive needs, insufficient menstrual and sexual health support, restricted autonomy in women’s decision-making, poor spousal communication, financial constraints, and low participation in sexual and reproductive health programs (Birhan *et al.*, 2018; Sharma *et al.*, 2018; World Health Organisation, 2019; Diamond-Smith *et al.*, 2024).

Understanding reproductive rights enables husbands to plan and intervene in maternal healthcare, use modern scientific developments like contraception, prevent diseases (STIs and HIV/AIDS), and promote pleasant sexual lives (Mohammed *et al.*, 2021b). Conversely, limited husbands’ knowledge has a significant detrimental impact on the implementation of their partners’ reproductive health rights. These gaps lead to low health service usage, lack of access to reproductive-related knowledge and utilization of scientific progress, such as contraception and other reproductive rights, resulting in a high burden of maternal morbidity and mortality (Germain, 2014; Tadesse *et al.*, 2020a).

Generally, despite the influence of men on their partners’ use of reproductive rights and the associated consequences, the level of husbands’ knowledge and related factors has not been achieved as required, both nationally and in the study setting. As a result, the importance of this study was to help plan good interventions for women’s reproductive health rights against poor reproductive health services to achieve better health outcomes. Therefore, this study aimed to determine the level of knowledge regarding reproductive rights and factors that influence them among husbands in Wolkite town, central Ethiopia.

## Methods

### Study area and period

The research was conducted in Wolkite town from March 15 to April 30, 2023. Wolkite town is the administrative capital of the Gurage Zone, located in the Central Ethiopia Regional State. It is situated 158 kilometers south of Addis Ababa on the route to Jimma town. Two rural and six urban Kebeles serve as the town’s administrative hubs. It has one referral and teaching hospital, three health centers, and 11 private clinics. The estimated population was 79,987, according to the 2007 Ethiopian census projection for 2019/2020, with 15,997 households (Gelgelu, 2018; Bisrat, 2024).

### Design and population of the study

A community-based cross-sectional study was conducted in Wolkite town. The source populations were all husbands residing in Wolkite town who had partners of reproductive age (15–49 years). All husbands who had reproductive-age partners during the study period were included in the study. Husbands who were critically ill were excluded from the study.

### Study population

All husbands residing in Wolkite town who had reproductive-age partners (15–49 years old) during the study period.

### Study unit

A husband with a partner who is of reproductive age.

### Sample size determination

The sample size was calculated using a single population percentage formula based on frequency assumptions (husband’s knowledge of their partner’s sexual and reproductive rights (48.3%) from a study conducted in Harar city (Mohammed *et al.*, 2021b), 95% CI, 5% margin of error, 10% non-response rate, considering the design effect of 1.5, which yielded ***632***). Sample size determination for the second objective: the factors for husbands’ knowledge of partners’ reproductive rights were obtained from the same study and calculated by Epi Info 7 stat calculation, with assumptions of 95% CI, 80% power, and exposure to the unexposed ratio of 1:1. Since the sample size for the single population proportion (632) was greater than the sample for associated factors (115, 201, and 320), the ultimate sample size for this study was 632.

### Sampling procedures

**Figure 1.**
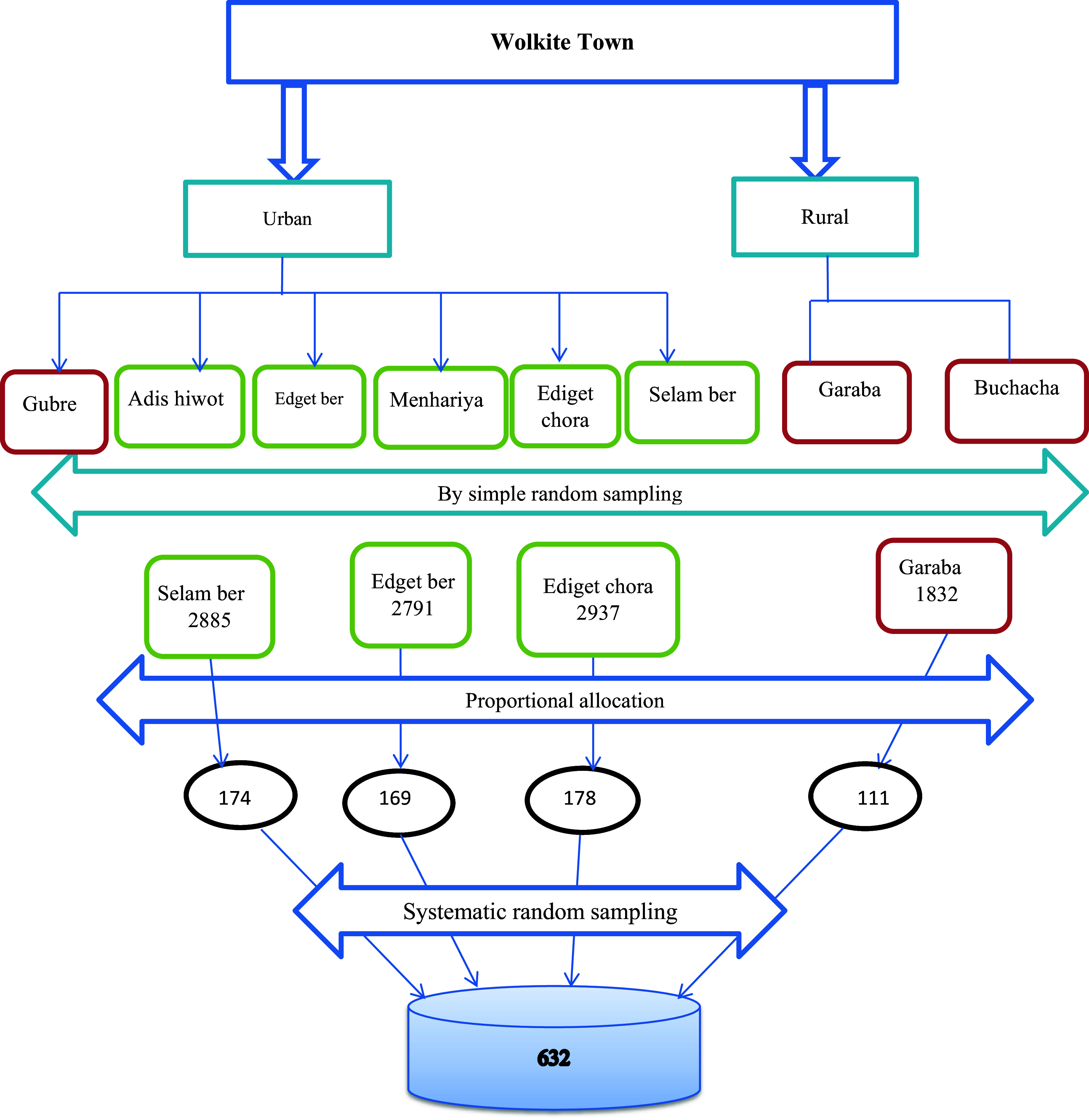
Schematic presentation of sampling procedure of study participants at Wolkite town, central Ethiopia, 2023.

The town’s Kebeles were first divided into urban and rural categories. Eight Kebeles, six urban and two rural, are in the city. Then, using the basic random selection approach, one out of two rural and three out of six urban Kebeles were chosen from each stratum. The study used a family folder of kebele administration households with reproductive-age women as a sampling frame, which is regularly updated. The first household was selected using a lottery method between one and K, where *K* = 10,445/632 = 16. The study participants were chosen by keeping the class interval at sixteen and including all eligible husbands until the required sample size was reached. A lottery method was employed to choose one eligible participant from households with more than one eligible member (Figure 1 **).**

### Study variables

**Outcome variable:** Husbands’ knowledge of partners’ reproductive rights.

**Predictor variables:** Socio-demographic characteristics such as age, religion, residence, educational level, occupation, family size, and discussion on reproductive issues. Health service and information-related: reproductive service user experience, mass media use, access to the reproductive education program, type of health facility present nearby, means of transportation, and distance to reach a health facility.

### Operational definitions

***Women’s reproductive rights**:* the right of women to make free decisions and choices on the twelve basic human rights (Germain, 2014).

***Husband’s knowledge**:* 11 questions are adapted to determine husbands’ understanding of their partners’ reproductive rights, and each correct answer has a score of one, while each incorrect response or ‘do not know the answer’ has a score of zero. Based on the summative scores from questions designed to determine the participants’ overall knowledge of women’s reproductive rights, they were categorized as having poor or good knowledge (Adinew *et al.*, 2013b; Tadesse *et al.*, 2020a).

***Good knowledge:*** Husbands who scored equal to the mean value or higher on correct knowledge questions were considered to have good knowledge of their partners’ reproductive health rights. Those who scored less than the mean value, on the other hand, were considered to have **poor knowledge** of their partners’ reproductive health rights.

### Data collection tool and procedures

A structured questionnaire administered by an interviewer was adapted from other literature created for a similar purpose by various authors (Adinew *et al.*, 2013b; Tadesse *et al.*, 2020a). The questionnaire contained three parts: socio-demographic characteristics, health service and information-related factors, and knowledge of reproductive rights. Eight health extension workers and two supervisors (BSc midwives) from Wolkite town were selected for data collection based on their experience and fluency in the local languages. The validity and reliability of the tool were checked. Content validity was assessed by field experts and professionals for relevance, clarity, and completeness, while reliability was evaluated using Cronbach’s alpha, which was 0.86. Data collectors conducted face-to-face interviews using a structured and pretested Amharic questionnaire. The first participant was selected by lottery, and subsequent participants were chosen at a class interval of sixteen among eligible husbands on the first day. In households with more than one eligible member, a lottery was used to select one participant to ensure equal probability of inclusion. Selected husbands were interviewed at their homes, with up to three visits if initially unavailable. On average, each interview lasted approximately 25 to 30 minutes. Husbands who could not be reached after these attempts were considered non-respondents.

Two days of training were given to supervisors and data collectors on the purpose, objectives, confidentiality, rights of respondents, informed consent, and interviewing techniques. After that, data collectors gave study participants an explanation of the purpose, objectives, confidentiality, and their right to decline or withdraw. After all, study participants gave their signed and written consent, and data collectors used a pre-tested questionnaire to gather data from eligible study participants and set up a convenient time for a follow-up when respondents were unavailable at home. The principal investigator and supervisors follow the data collection procedure on each day of the study.

### Data quality control

The principal investigator provided comprehensive training to data collectors and supervisors on data collection procedures, ethical considerations, data validity risks, and each question included in the study to ensure data quality.

The questionnaire was pretested on a 5% sample size at Addis Hiwot Kebele before the actual data collection period to assess its simplicity, sequence, coherence, clarity, and time to complete. Later, any ambiguity, complex words, and differences in comprehension were revised based on pretest experience. Participants were given a detailed explanation of the study’s purpose, procedure, confidentiality, and benefits to obtain informed consent and reliable data.

Finally, after ensuring that the data was complete and properly coded with a unique identification number, each response was entered into the software for analysis. Epidata was used for data entry because it has an error detection mechanism built in. To ensure data consistency, two separate data clerks double-entered the information. Data was stored in the form of a file in a secure location where only the principal had access to it.

### Statistical analysis

Data entry was done using Epi Data version 4.1. The entered data was checked and exported to SPSS version 26 for data analysis. Different frequency tables, graphs, and descriptive summaries were used to describe the study variables. In the bivariable analysis, crude odds ratios (COR) with 95% confidence intervals were computed to identify candidate variables for the multivariable analysis. Binary logistic regression was used to assess independent predictors of husbands’ knowledge of their partners’ reproductive rights with the formula of Log (p*i*/1-p*i*) = β0+β1X1*i*+β2X2*i*+⋯+β*k*X*ki*. where pi is the probability that a husband has good knowledge (Y*i* = 1), β_0_ is the intercept, β1, β2,…,β*k* are regression coefficients, and X_1*i*_, X_2*i*_, …, X_*ki*_ are independent variables. Logistic regression models the probability of the outcome, and the coefficients are estimated using maximum likelihood without a separate error term. Independent variables with a significance level of *p*-value < 0.25 at 95% CI in bivariable analysis and which were fit for the model of regression were retained for inclusion into the multivariable logistic regression to control all possible confounders.

Multicollinearity was checked to see the linear correlation among the associated independent variables by using the variance inflation factor (VIF) and standard error. VIF of >10 or standard error of >two was considered suggestive of the existence of multicollinearity. No multicollinearity was detected during the analysis. For all independent variables, the multicollinearity effect was checked by collinearity diagnostic statistics via VIF and tolerance test with a maximum value of 2.34 and a minimum value of 35.6%, respectively.

In multivariable analysis, the multivariable logistic regression model was used to control the confounders. The Hosmer-Lemeshow goodness-of-fit test was done to check for model fitness with a *p*-value of 0.542, which indicates the model was fitted. Adjusted Odds Ratio (AOR) with 95% CI was estimated to show the strength of association between the independent variables and the dependent variable after controlling for the effects of confounders. The results were considered statistically significant at a *p*-value < 0.05. Finally, tables, graphs, and narration were used to present the findings.

### Ethical approval and consent to participate

Ethical clearance was obtained from the Institutional Health Research Ethics Review Committee (IHRERC) of Bahir Dar University, with reference number IHRERC/008/23. The study was conducted based on the ethical standards of the Declaration of Helsinki. The ethical letter was given to the Gurage Zone health office and the Wolkite town health office to get permission for the data collection process. The purpose of the study and their right to refuse were explained to the study participants, and informed written consent was taken. Coding was used to eliminate names and other personal identification of respondents throughout the study process to ensure participant confidentiality.

## Results

Among a total sample of 632 study participants, 628 were interviewed and gave a response rate of 99.4%, and the results were presented as follows under subheadings.

### Socio-demographic characteristics of the study participants

The minimum and maximum ages of the respondents were 20 years and 60 years, respectively, with a mean age of 36.94 ± 7.67 years. The minimum and maximum ages of the partners were 18 years and 45 years, respectively, with a mean age of 30.1 ± 6.16 years. Regarding educational status, about one hundred sixty-four (26.1%) respondents have no formal education **(**Table 1).

**Table 1.** Socio-demographic characteristics of husbands in Wolkite town, Central Ethiopia, 2023 (*n* = 628)

Variables	Category	Frequency (*n*)	Percent (%)
Age of the respondent (years)	≤25 years	40	6.4
	26-35	219	34.9
	35 and above	369	58.8
Age of the partner (years)	≤25 years	185	29.5
	26–35	309	49.2
	35 and above	134	21.3
Religion	Orthodox	264	42.0
	Muslim	232	36.9
	Catholic	47	7.5
	Protestant	77	12.3
	Others	8	1.3
Occupational status	Employed	218	34.7
	Merchants	282	44.9
	Farmers	107	17.0
	Others	21	3.3
Residence	Urban	517	82.3
	Rural	111	17.7
Family size	≤3	228	36.3
	Four and above	400	63.7
Educational status	No formal education	164	26.1
	Read and write	136	21.7
	Primary education	95	15.1
	Secondary education	69	11
	Above secondary education	164	26.1

Other* wake feta, Jova other** Daily laborer, driver.

### Health service and information-related factors

Of the respondents, 593 (94.4%) utilized mass media. When calculating the time it takes to get from their homes to medical facilities, 79 people (12.0%) take longer than 30 minutes. In addition, 376 (59.9%) and 274 (43.6%) of the husbands have experience with using reproductive services and have available reproductive education programs, respectively. Similarly, 433 (68.9%) of the respondents have a discussion on reproductive health-related matters with their partners (Table 2).

**Table 2.** Health service and information-related factors among the husbands in Wolkite town, Central Ethiopia, 2023 (*n* = 628)

Variables	Category	Frequency (*n*)	Percent (%)
Mass media use	Yes	593	94.4
	No	35	5.6
Type of mass media (*n* = 593)	Radio only	97	15.4
	Television only	202	32.2
	Other mass media	8	1.3
	More than one	286	45.5
The nearby health facility	Hospital	273	43.5
	Health center	201	32.0
	Health post	154	24.5
Means of transport	Vehicles	380	60.5
	Other than the vehicle	248	39.5
Discussion	Yes	433	68.9
	No	195	31.1
Experience in using reproductive services	Yes	376	59.9
	No	252	40.1

Other means of transport are by foot and animal.

### Husbands’ knowledge of partners’ reproductive health rights

**Figure 2.**
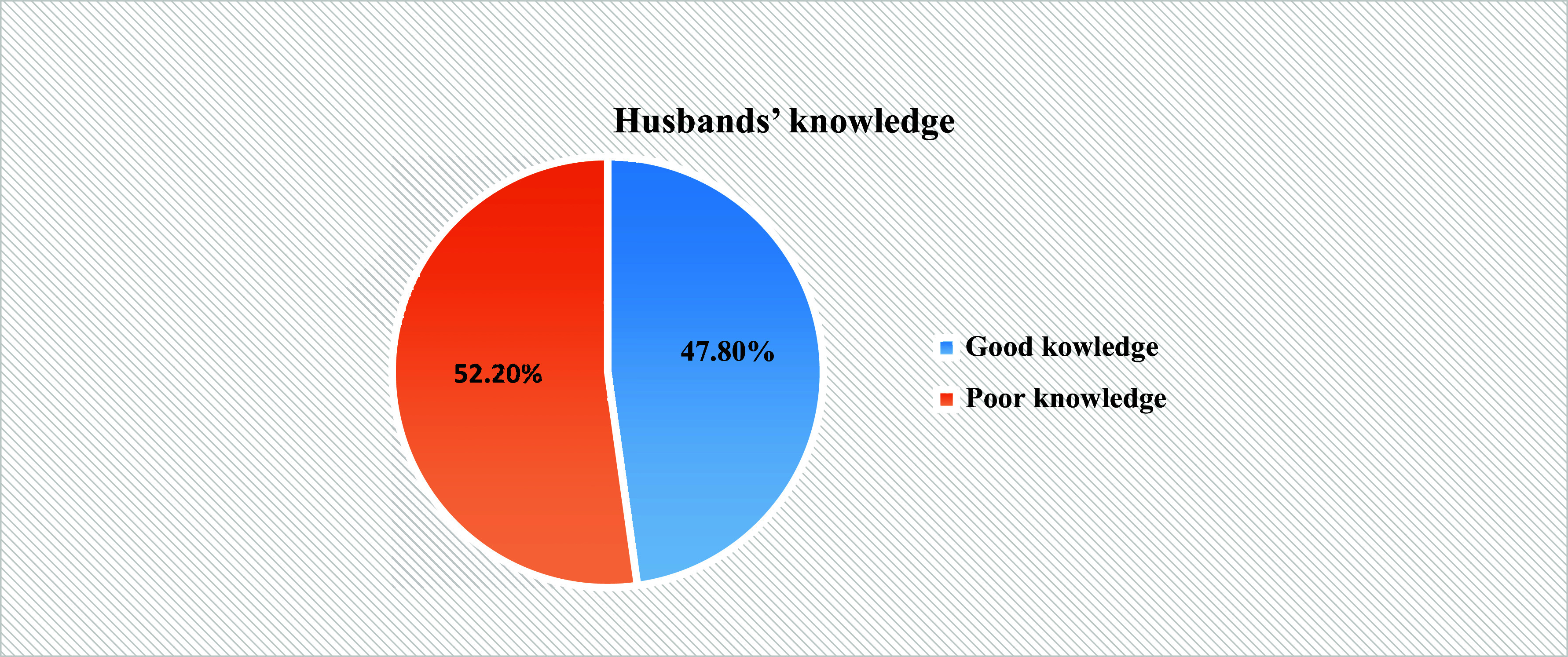
Knowledge level of reproductive health rights among husbands in Wolkite town, central Ethiopia, from March 1 to April 30, 2023, GC.

The prevalence of husbands’ good knowledge of partners’ reproductive rights was 47.8% (95% CI: 43.8, 51.8). About half of the respondents were aware that married women have the complete right to obtain all reproductive health care without their husbands’ permission **(**Figure 2 **).** Almost one-fourth of the husbands (24.4%) were aware that married women have a right to the confidentiality of their reproductive health information.

### Factors associated with husbands’ knowledge of their partners’ reproductive rights

In this study, candidate variables were selected with a *p*-value < 0.25 in bivariate analysis.

Among the variables, the age of the respondent, partners’ age, educational status, family size, nearest health facility, means of transport, availability of RH program, discussion with RH matters, and experiences of using RH services were candidate variables for the multivariable analysis.

Among the candidate variables, the age of the respondent, the educational status of the respondent, discussion with RH matters, and means of transport were statistically significant with the outcome variable. Husbands between the ages of 25 and 35 were 2.7 times more likely to be knowledgeable about their partners’ reproductive rights than those under the age of 25 (AOR: 2.7; 95% CI: 1.10, 6.60).

Husbands who can read and write were two times more likely to have good knowledge when compared to those with no formal education (AOR: 2.4; 95% CI: 1.3, 4.3). The odds of having good knowledge about partners’ reproductive rights among husbands who have primary education were 5.98 times more likely as compared with those with no formal education (AOR: 5.98; 95% CI: 3.1, 11.4). The husbands who had learned secondary school were two times more likely to have good knowledge as compared to those with no formal education (AOR: 2.1; 95% CI: 1.0, 4.3). Moreover, husbands who had completed above secondary education were eight times more likely to have good knowledge about their partners’ reproductive rights than those without formal education (AOR: 8.0; 95% CI: 4.3, 15.2).

Husbands having good knowledge who discussed reproductive health matters with their partner were three times more likely to do so as compared with those who had not discussed reproductive health matters (AOR: 3.2; 95% CI: 2.0, 5.1). Respondents who have used vehicles as a means of transport were three times more likely to have good knowledge as compared with those who use other means of transport (AOR: 3.3; 95% CI: 2.2, 4.9) **(**Table 3).

**Table 3.** Factors associated with husbands’ knowledge of their partner’s reproductive rights in Wolkite town, Central Ethiopia, 2023 (*n* = 628)

Category	Knowledge status		COR (95% CI)	AOR (95% CI)	*p*-Value
	Good	Poor			
Age of respondent					
Less than 25 years	10	28	1	1	
26–35	145	76	5.34 (2.47,11.59)	2.7 (1.1,6.6)	< 0.001
35 and above	145	224	1.81 (0.86,3.84)	1.4 (0.6,3.3)	0.23
Partners’ age					
Less than 25 years	107	78	1	1	
26–35	151	158	0.69 (0.48,1.06)	0.9 (0.5,1.7)	0.1
Above 35	42	92	0.33 (0.21,0.53)	0.7 (0.3,1.5)	0.33
Educational status					
No formal education	28	136	1	1	
Read and write	54	82	3.19 (1.88,5.45)	2.4 (1.3,4.3)	< 0.001
Primary	54	41	6.39 (3.60,11.36)	5.98 (3.1,11.4)	< 0.001
Secondary	32	37	4.20 (2.25,7.84)	2.1 (1.0,4.3)	< 0.001
Above secondary	132	32	20.3 (11.43,35.11)	8.0 (4.3,15.2)	< 0.001
Family size					
Less than three	75	153	1	1	
Four and above	225	175	2.62 (1.87,3.68)	1.09 (0.69,1.71)	0.21
Nearest health facility					
Hospital	173	100	3.82 (2.51,5.82)	1.60 (0.96,2.8)	0.07
Health center	79	122	1.43 (0.92,2.23)	0.9 (0.5,1.6)	0.13
Health post	48	106	1	1	
Means of transport					
Vehicle	268	282	1.37 (0.84,2.21)	3.3 (2.2,4.9)	0.02
Other means of transport	32	46	1	1	
Availability of the RH program					
Yes	154	120	1.83 (1.33,2.52)*	0.9 (0.6,1.4)	0.12
No	146	208	1	1	
Discussions with RH matters					
Yes	259	174	5.59 (3.76,8.29)	3.2 (2.0,5.1)	< 0.001
No	41	154	1	1	
Experiences of using RH services					
Yes	200	176	1.73 (1.25,2.39)*	1.04 (0.68,1.60)	0.08
No	100	152	1	1	

CI: Confidence interval, COR: Crude odds ratio, AOR: Adjusted odds ratio.

## Discussion

The prevalence of husbands’ good knowledge of partners’ reproductive rights was 47.8% (95% CI: 43.8, 51.8). This finding was aligned with a study done in Harar, which found that 48.3% of husbands have good knowledge of their partners’ reproductive rights (Mohammed *et al.*, 2021b). This consistency may have something to do with the study population, study design, and study period. Furthermore, it could be because government intervention tactics are the same throughout the country. To achieve universal primary health care coverage by 2009, the Ethiopian government initiated a health extension program in 2003. Health extension professionals are employed by this program to raise awareness and prevent disease and to promote health and prevent illness. As a result, health extension professionals’ work involves raising awareness of reproductive rights. (Workie and Ramana, 2013). Likewise, there are established one-to-five collaborations among the communities and community health development agencies as ways to share health-related issues in both study areas. Through this network, the husbands can exchange knowledge with other people.

This finding was greater than the study conducted in Nepal, which revealed that 9.1% of husbands have good knowledge of their partners’ reproductive rights (Yadav *et al.*, 2016). These differences might be due to the assessment tool utilized, socio-economic status, study period variation, and government intervention strategies variation between the two countries (Nepal *et al.*, 2023). However, the results of this study were less than the study done in South Africa, which was 56.1%, and Ghana, which was 53.8% (Yendaw *et al.*, 2015; Govender *et al.*, 2019). This discrepancy might be due to variation in educational level; all respondents in South Africa and Ghana were university students, but in this study, most respondents were below secondary educational level. This is because students who attended higher education could have the potential to analyze and understand reproductive health rights in advance (Adjiwanou *et al.*, 2018).

Husbands between the ages of 25 and 35 were 2.7 times more likely to be knowledgeable about their partners’ reproductive rights than those under the age of 25. This difference might be due to generational variations in access to information and exposure to reproductive health education. Husbands aged between 25 and 35 years are more likely to be active users of social and mass media platforms, which are increasingly used to disseminate information on sexual and reproductive health (Mohammed *et al.*, 2021a).

Husbands who can read and write are two times more likely to have good knowledge as compared with those with no formal education. The odds of having good knowledge about partners’ reproductive rights among husbands who have a primary education were six times higher than compared with those not educated. Husbands who attended secondary school were two times higher as compared as those with no formal education. Similarly, the odds of having good knowledge about partners’ reproductive rights among husbands who studied above secondary education were eight times higher as compared with those with no formal education (Jemberie *et al.*, 2024a).

This might be related to the increase in the educational status of the husbands, increasing the act beyond the cultures and beliefs. In addition, it might be due to information sharing, the difference in exposure, and communication on reproductive health rights through their educational advancement (Jemberie *et al.*, 2024a). Similarly, those who have attended higher education could have the potential to analyze and understand reproductive health rights in advance, and the fact that education, considered the single most important socio-economic characteristic, positively affects reproductive health knowledge and behaviors (Adjiwanou *et al.*, 2018).

Husbands having good knowledge who discussed reproductive health matters were 3 times more likely as compared with those who had not discussed reproductive health matters with their partner. This research finding aligns with the study employed in Wolayita Sodo and Adet Tana Haik (Ayalew *et al.*, 2019). It was also consistent with the study done at Harar, which showed that partners who openly discussed their reproductive health were twice as likely to be knowledgeable about their partners’ reproductive rights as those who did not have a discussion (Mohammed *et al.*, 2021b).

This may have something to do with raising awareness of the rights of reproductive health. People will exchange thoughts, opinions, and facts about issues about reproductive health through dialogue (Adugnaw *et al.*, 2011; Gebreselassie *et al.*, 2019a). This is explained by the fact that sharing experiences during a conversation might help people learn more about reproductive rights (Gebresilassie *et al.*, 2019b; World Health Organization, 2019).

Respondents who have used vehicles as a means of transport were 3.3 times more likely to have good knowledge as compared with those who used other means of transport. It might be due to using a vehicle as a means of transport being related to the economic status of the respondents. Most of the respondents who preferred to walk on foot and use animals were those who had no means to pay the transportation fee (Sidqia *et al.*, 2025). Even though health facility access can result in changing the perception, belief, health norms, and practices of individuals (Tadesse *et al.*, 2020b; Jemberie *et al.*, 2024a), they may not prefer to visit health facilities for fear of or lack of transportation fees, which hinder them from knowing their partners’ reproductive rights, particularly the rural residents (Grulich *et al.*, 2025).

## Strengths and limitations of the study

### Strength of the study

The strength of this study includes various categories of men, and the sample was taken at random. Hence, the results of this study can represent the population. In this study, potential biases were minimized by using clear objectives, pretested and valid questionnaires, training provided for data collectors and supervisors, a random sampling method, an ideal sample size, and statistical adjustments (multivariable regression) to account for confounding variables. Finally, ethical guidelines were implemented to ensure unbiased participants.

### Limitations of the study

This study was based on the survey data that was self-reported and may be influenced by potential bias from social desirability, since men may report responses that are more acceptable. Since a cross-sectional design was used, the cause-and-effect relationship could not be established. Therefore, research like a longitudinal study and a qualitative study should be conducted to identify factors and gain a deeper understanding of men’s knowledge regarding their spouses’ reproductive health rights, using a large sample size.

## Conclusion

In Wolkite Town, less than half of the husbands (47.8%) had good knowledge about their partners’ reproductive health rights. Husbands’ age, educational status, engagement in discussions about reproductive health issues, and access to transportation were significantly associated with the husbands’ knowledge of their partners’ reproductive health. Incorporating reproductive health rights into community women’s affairs and early childhood education should increase awareness of these rights and promote reproductive rights discussions between couples. Improving husbands’ knowledge of reproductive rights is crucial to ensure gender equality and to promote informed decision-making within families and communities. It makes a great contribution to healthy maternal and child health outcomes.

## Recommendations

The following recommendations are based on the findings: Integrate reproductive health rights into education programs with simple, culturally relevant information aimed at men with low literacy. Develop age-appropriate reproductive health education by engaging both younger and older men through youth clubs, social media campaigns, community meetings, and religious programs, respectively. Encourage open communication between spouses by organizing culturally acceptable discussion forums. Additionally, train male community leaders and health extension workers to serve as role models and educators on reproductive rights. Promote male participation in reproductive health policies and programs at the woredas and kebele levels. Furthermore, strengthen the role of women’s affairs offices to involve men in promoting gender equality and shared decision-making.

In addition, improving transportation infrastructure and affordability to ensure equitable access to health services, particularly in rural areas. Encourage peer education programs where couples (the husbands and their partners) could discuss their reproductive health openly. Improve the utilization of mass media (radio, TV) and social media platforms to disseminate information on reproductive rights, targeting young men. Policymakers should institutionalize male engagement as a cross-cutting component in all reproductive health policies and strategic frameworks. Furthermore, it is recommended to strengthen intersectoral collaboration among the Ministry of Health, the Ministry of Education, and Women and Social Affairs to ensure that reproductive rights education is delivered through a coordinated and multi-sectoral approach.

## Data Availability

Data that support the findings are available from the corresponding author upon a reasonable request. The data are not publicly available due to privacy or ethical restrictions.
